# Comment on “Obituary: Professor Andreas Gal”

**DOI:** 10.1155/humu/8991628

**Published:** 2026-09-10

**Authors:** Christian Kubisch, Kerstin Kutsche

**Affiliations:** ^1^ Institute of Human Genetics, University Medical Center Hamburg-Eppendorf, Hamburg, Germany, uke.de

It is with great sadness that we announce the passing of Professor Andreas Gal, who died on May 20, 2026. He served as an editor of *Human Mutation*; his editorial work was distinguished by integrity, fairness, and an unwavering commitment to supporting researchers from around the world.

Andreas Gal was born on September 18, 1947, in Debrecen, Hungary. He studied medicine at the University of Szeged from 1966 to 1972 before beginning his scientific career at the prestigious Institute of Genetics of the Hungarian Academy of Sciences in Szeged, where he worked from 1973 to 1983. Following a brief appointment at the Institute of Human Genetics in Freiburg, Germany, he became head of the Laboratory of Molecular Human Genetics and DNA Diagnostics at the Institute of Human Genetics, University of Bonn, in 1985. In this role, he emerged as one of the pioneers of molecular genetics diagnostics in Germany. In 1990, together with his appointment to a professorship funded by the Schilling Foundation, he became head of the Research Group for Molecular Human Genetics and DNA Diagnostics at the Institute of Human Genetics, Medical University of Lübeck, Germany. In 1994, he was appointed full professor and director of the Institute of Human Genetics at the University Medical Center Hamburg‐Eppendorf, Hamburg, Germany.

Andreas Gal is particularly renowned for his contributions to the identification of disease‐causing genes and the elucidation of molecular mechanisms underlying both rare and more common inherited disorders. His research encompassed a broad spectrum of genetic diseases, including retinitis pigmentosa, Norrie disease [1], Usher syndrome, and systemic lysosomal disorders such as the mucopolysaccharidoses [2–4] and Fabry disease [5]. Among his most influential publications are seminal studies on the genetic basis of *RPE65*‐associated Leber congenital amaurosis [6], *RDH12*‐associated retinal dystrophy [7], retinitis pigmentosa, *MERTK*‐associated retinal disease [8], *CDH23*‐associated Usher syndrome [9], and autosomal recessive posterior microphthalmia caused by pathogenic variants in *PRSS56* [10].

In recognition of his outstanding scientific achievements, Andreas Gal received numerous prestigious awards. These included the “Fight Against Blindness” research award from the German and Swiss Retinitis Pigmentosa Associations in 1989; the recognition award from the Alcon Research Institute (Fort Worth, Texas) in 2006 for outstanding contributions to vision research; the recognition award from Retina International in 2012 for exceptional achievements in the field; and, in 2013, the Honorary Medal of the German Society of Human Genetics in recognition of his distinguished contributions to human genetics. In 2017, he delivered the prestigious François Lecture at the meeting of the International Society for Genetic Eye Diseases and Retinoblastoma (ISGEDR) in Leeds, United Kingdom. These honors reflect not only the high international esteem in which he was held by the scientific community but also the lasting impact of his research on our understanding of inherited retinal diseases and its contribution to the development of novel therapeutic approaches.

Andreas Gal′s extraordinary dedication to the research of inherited diseases, together with his unwavering commitment to educating and mentoring generations of physicians and scientists, established him as one of the leading academic figures of his generation in human genetics. He will be greatly missed. His scientific legacy, however, will continue to shape our field for many years to come.

## Conflicts of Interest

The authors declare no conflicts of interest.
